# The association of dietary and lifestyle indices for hyperinsulinemia with odds of non-alcoholic fatty liver disease in Iranian adults: a case–control study

**DOI:** 10.1186/s40795-023-00675-3

**Published:** 2023-01-20

**Authors:** Aref Momeni, Rouhollah Haghshenas, Soodeh Razeghi Jahromi

**Affiliations:** 1 Department of Physical Education and Sport Science, Humanity Faculty, Semnan University, Semnan, Iran; 2 Department of Sport Sciences, Faculty of Humanities, Semnan University, Semnan, Iran; 3 Department of Clinical Nutrition and Dietetics, Faculty of Nutrition and Food Technology, Shahid Beheshti University of Medical Sciences, Tehran, Iran

**Keywords:** Physical activity, Lifestyle, Dietary pattern, Insulinemic indices, Adults, Non-alcoholic fatty liver disease

## Abstract

**Background:**

Evidence on the association of insulinemic effects of dietary pattern and other lifestyle factors with the odds of non-alcoholic fatty liver disease (NAFLD) are limited. In the current study, we aimed to examine the association of the empirical dietary index for hyperinsulinemia (EDIH) and empirical lifestyle index for hyperinsulinemia (ELIH) index with the NAFLD odds in the adult population.

**Methods:**

In the current case–control study, 120 cases of NAFLD and 240 controls aged 20–60 years were included. The ultrasonography test was used to determine NAFLD. We used a validated food frequency questionnaire to collect dietary data of individuals and determine the scores of EDIH. Also, we determined the ELIH score based on diet, body mass index, and physical activity. The odds ratio (OR) of NAFLD was calculated using logistic regression test across EDIH and ELIH tertiles.

**Results:**

The mean ± SD age of subjects (53% men) were 41.8 ± 7.5 years. In the age and sex-adjusted model, there is a significant association between a higher ELIH score and higher odds of NAFLD (OR = 2.74;95%CI:1.51–4.96,P_trend_ = 0.001). Also, based on the multivariable-adjusted model, after controlling for age and sex, smoking, SES, and dietary intake of energy a remarkable positive association was observed between the higher score of ELIH and the odds of NAFLD (OR = 2.70; 95%CI:1.46–5.01,P_trend_ = 0.002). However, there is no significant relationship between the higher score of EDIH and NAFLD odds.

**Conclusions:**

Our results showed that the high insulinemic potential of lifestyle, determined by the ELIH score, can be related to an increased NAFLD odds. However, no significant association was found between higher EDIH score and odds of NAFLD.

## Background

Non-alcoholic fatty liver disease (NAFLD) is recognized as a global public health problem and is the leading cause of chronic liver disease; it is estimated to affect 25% of the world's population [1]. Convincing evidence shows a strong relation between NAFLD and the odds of developing multiple extrahepatic complications such as diabetes, cardiovascular disease, chronic kidney disease, and other chronic diseases [2].

Although the pathophysiology of NAFLD remains largely unclear, it seems that hyperinsulinemia and insulin resistance (IR) are involved in NAFLD development through enhancement of fatty acid synthesis in the liver would reduce hepatic fatty degeneration [3]. In a 5-year follow-up cohort study, high baseline and continuously increasing fasting insulin levels were the independent determinants for future development of NAFLD [4]. In another study, Manchanayake et al. observed that postprandial hyperinsulinemia is universal in nondiabetic patients with NAFLD [5]. Recently, Tabung and his colleagues proposed two indices, including the empirical dietary index for hyperinsulinemia (EDIH) and the empirical lifestyle index for hyperinsulinemia (ELIH), that predict the potential of diet and lifestyle for hyperinsulinemia [6]. EDIH was developed based on a series of dietary food groups with positive and negative correlations with hyperinsulinemia. ELIH index considers the effect of body mass index (BMI) and physical activity as two important lifestyle factors in hyperinsulinemia besides the dietary intake. Studies investigated the association of these indices with various diseases, including hyperinsulinemia [7], IR [7], diabetes [8–10], NAFLD [11, 12], and cancers [13, 14].

Higher adherence to EDIH score was associated with a higher risk of diabetes incidents [9, 10] and weight gain [15] among US populations, however, two previous studies on Iranian adults showed no significant association between EDIH with the incidence of IR, hyperinsulinemia [7], and diabetes [8].

To the best our knowledge, just two previous studies investigated the relation between EDIH and NAFLD [11, 12]. In a cross-sectional study, it has been reported that individuals who had higher adherence to EDIH increasingly had a higher prevalent odds of hepatic steatosis and advanced fibrosis by 17% and 74%, respectively [12]. Also, in a case–control study higher score of EDIH was associated with higher odds of NAFLD [11]. Although no previous study assessed the ELIR and NAFLD relationship, higher adherence to an ELIR score showed a significant association with hyperinsulinemia [7] and diabetes incident [8].

Considering that studies conducted on the association of EDIH and ELIH with NAFLD are scarce, and findings are inconsistent, the present study was conducted to investigate this association in a case–control study among Iranian adults.

## Material and methods

### Study design and population

The present case–control study was conducted in Tehran's gastroenterology and diabetes clinic. Patients who were referred to evaluate for their probability of NAFLD by an Ultrasonography (USG) test because of having an abnormal or slight elevation in liver enzymes or being at risk of metabolic syndrome or having metabolic syndrome, etc., were assessed for eligibility criteria of the present study. Individuals who were diagnosed with NAFLD by USG guidance and physician's confirmation were included in the case group and other people who were not identified as having the disease were included in the control group.

We included 20–60 years old participants with willing to cooperate in the study without a history of renal and hepatic diseases (Wilson’s disease, autoimmune liver disease, hemochromatosis, virus infection, and alcoholic fatty liver(, cardiovascular disease (CVD), diabetes, malignancy, thyroid disorder, and autoimmune, not following a specific diet (due to a particular disease or weight loss) and not using potentially hepatotoxic or steatogenic drugs.

The sample size calculation was conducted using the G power software version 3.1. Considering the odds ratio (OR) of NAFLD by 1.35 for the highest versus lowest tertile of EDIH using two previous studies [11, 12], type I error of 5%, and study power of 80% (β = 0.20), and the ratio of controls to cases as 2, we needed a sample of 95 NAFLD patients and 190 controls. However, we recruited 120 patients with NAFLD and 240 controls to keep track of any possible drop-outs. Individuals in case and control groups matched on age ± 2 years.

### Dietary assessment

Dietary intakes were collected using a validated 168-item semi-quantitative FFQ among Iranian adults [16]. Participants were asked to report their average dietary intake during the previous year by choosing one of the following choices: never or less than once a month, 3–4 times per month, once a week, 2–4 times per week, 5–6 times per week, once daily, 2–3 times per day, 4–5 times per day, and six or more times a day. Portion sizes of each food item were converted into grams by using standard Iranian household measures [17]. Daily energy and nutrients intakes for each participant were computed using the United States Department of Agriculture’s (USDA) Food Composition Table (FCT) [18]. The Iranian FCT was used for some traditional foods that are not listed in USDA FCT.

### The calculation of indices

Indices were calculated using the method introduced by Tabung et al. [6]. For EDIH 15 food items were categorized into two groups according to their potential to induce or reduce hyperinsulinemia. Those with positive associations were processed meat (sausage), red meat (beef or lamb), fish (canned tuna or fish), margarine, poultry (chicken or turkey with or without skin), French fries, high-energy beverages (cola with sugar, carbonated beverages with sugar, fruit punch drinks), tomatoes, low-fat dairy products (skimmed or low-fat milk and yogurt) and eggs. Furthermore, food items with inverse association were coffee, green leafy vegetables (cabbage, spinach, or lettuce), whole fruits, and high-fat dairy products (whole milk, cream, cream cheese, and other cheese). The EDIH score was calculated as follows^*^:

EDIH = (Red meat * 0.250 + processed meat * 0.199 + margarine * 0.054 + poultry * 0.183 + butter * 0.094 + French fries * 0.581 + other fish * 0.172 + high-energy beverages * 0.104 + tomatoes * 0.095 + low-fat dairy * 0.025 + eggs * 0.124 + coffee * -0.035 + whole fruits * -0.029 + high-fat dairy products * -0.046 + green leafy vegetables * -0.055) / 1000. ^*^All food groups were included as serving per day.

The ELIH encompasses 11 dietary and lifestyle factors, including BMI, margarine, butter, red meat, and fruit juice (apple juice, cantaloupe juice, orange juice, or other fruit juice) with a positive association and coffee, whole fruit, physical activity, high-fat dairy products, snacks (potato chips, corn chips or popcorn, crackers, and Cheetos) and salad dressing with the inverse association. The ELIH score was calculated as follows^*^:

ELIH: (Body mass index (kg/m2) * 0.051 + margarine * 0.041 + butter * 0.058 + red meat * 0.089 + fruit juice * 0.042 + coffee * -0.020 + whole fruits * -0.029 + physical activity (MET-h/week) * -0.001 + high-fat dairy products * -0.054 + snacks * -0.024 + salad dressing * -0.059) / 1000. ^*^All food groups were included as serving per day.

### Physical activity and anthropometric measurements

Physical activity was recorded using the International Physical Activity Questionnaire (IPAQ) [19] during a face-to-face interview, and it was expressed as Metabolic Equivalents per week (METs/week) [20, 21]. Participants’ weight was recorded by trained dieticians using a standard digital Seca scale (made in Germany), wearing minimum clothes and without shoes and the nearest 100 g. Height was measured in a standing relaxed shoulder position with no shoes using a tape meter mounted on the wall to the nearest 0.5 cm. BMI was calculated as weight (kg) divided by height in square meters (${\mathrm{m}}^{2}$).

## Assessment of other variables

The information on other variables, including age, sex, marital status, socioeconomic status (SES), and smoking status, was collected using a socio-demographic questionnaire. In the current study, we classified smoking into yes/no groups; ‘yes’ showed individuals who smoked cigarettes as daily or occasionally or ex-smokers, and ‘no’ defined the participants who are non-smokers [22]. We used three dichotomous variables, including family size (≤ 4, > 4 people), education (academic and non-academic education), and acquisition (house ownership or not) for the calculation of the SES score. For each of these variables, participants were given a score of 1 (if their family members were ≤ 4, were academically educated, or owned a house) or 0 (if their family members were > 4, or had non-academic education or leasehold property). Then, we summed the assigned scores as total SES scores (minimum SES score of 0 to a maximum score of 3). The total score of 0 or 1, 2, and 3 were determined as low, moderate, and high SES status, respectively.

## Statistical analysis

Statistical analysis was conducted using Statistical Package Software for Social Science, version 21 (SPSS Inc., Chicago, IL, USA). Kolmogorov–Smirnov’s test and histogram chart were used to assess the normality of variables. Participant characteristics and dietary data were reported as mean ± SD or median (25–75 interquartile range) and number (percent) for continuous and categorical variables, respectively. The independent sample t-test and chi-square were used to compare the continuous and categorical variables between cases and controls, respectively.

EDIH and ELIH were categorized into tertiles based on their cut-offs among controls, and general and dietary data across tertiles of EDIH and ELIH were reported. The P for the trend of the qualitative and qualitative variables across tertiles of EDIH and ELIH were calculated using linear regression (median values of EDIH or ELIH in each tertile as the independent variable and continuous variables as dependent variable) and chi-square test. Multivariable logistic regression was performed to assess the relationship between EDIH and ELIH with NAFLD odds. The OR with a 95% confidence interval (CI) of NAFLD across tertiles of EDIH and ELIH was reported. Our analyses was performed based on two models, including model 1 that was adjusted for age and sex, and multi-variable model that was adjusted for BMI (only for EDIH) and physical activity (only for EDIH), smoking, SES, and dietary intake of energy. P-values < 0.05 were considered statistically significant.

## Results

The current was conducted on 120 cases of NAFLD and 240 controls aged 20–60 years. Because of the incomplete dietary data (food frequency questionnaire (FFQ) less than 35 items) and under or over-reported daily energy intake (≤ 800 or ≥ 4200 kcal/d), 5 participants were excluded and replaced with new participants. The study population's mean ± SD age and BMI were 41.8 ± 7.5 years and 27.4 ± 2.2 kg/m^2^, respectively. 53.1 percent of participants were men. The median (interquartile) of EDIH and ELIH in participants was 0.15 (0.06 – 0.28) and 0.15 (-0.49 – 0.57), respectively.

Table 1 indicates the general characteristics and dietary intakes in all populations, case and control groups. The percentage of men and percentage of smoked individuals in the NAFLD group was higher than in the non-NAFLD group. Compared to non-NAFLD individuals, NAFLD patients had a significantly lower mean of physical activity and academic education and had higher mean BMI, energy intake, and poor socioeconomic status. Also, the scores of EDIH, ELIH, EDIR, and ELIR in the NAFLD group were higher than in the non-NAFLD group. However, the mean of age, dietary intakes of fats, carbohydrates, and protein were not significantly different between the two groups (P > 0.05).

**Table 1:** Baseline characteristics and dietary intakes of participants in both NAFLD and non-NAFLD groups^a,b^

Variables	Total (*n* = 360)	NAFLD (*n* = 120)	non-NAFLD (*n* = 240)	*P*-value
Age (year)	41.8 ± 7.5	41.4 ± 7.4	42.0 ± 7.6	0.508
Sex (male, %)	53.1	70.8	44.2	< 0.001
BMI (Kg/m^2^)	27.4 ± 2.2	28.5 ± 2.1	26.9 ± 2.1	< 0.001
Physical activity (MET.min/Wk.)	1482 ± 879	1263 ± 615	1592 ± 967	< 0.001
Smoking (yes, %)	5.6	10.8	2.9	0.002
Education level (bachelor and higher), n (%)	160 (44.4)	44 (36.7)	116 (48.3)	0.036
Socio-economic status (SES), n (%)				
Poor	138 (38.3)	35 (29.2)	103 (42.9)	0.008
Medium	134 (37.2)	48 (40.0)	86 (35.8)	
High	88 (24.4)	37 (30.8)	51 (21.3)	
Energy intake (kcal/day)	2257 ± 650	2406 ± 638	2182 ± 645	0.002
Carbohydrates (% of energy)	57.6 ± 7.0	57.8 ± 7.1	57.51 ± 7.0	0.721
Protein (% of energy)	13.8 ± 2.4	13.8 ± 2.6	13.8 ± 2.3	0.942
Fat (% of energy)	31.2 ± 6.8	30.9 ± 6.8	31.4 ± 6.8	0.490
Empirical Dietary Index for Hyperinsulinemia (EDIH)	0.15 (0.06—0.28)	0.18 (0.07—0.33)	0.15 (0.06—0.26)	0.047
Empirical Dietary Index for Insulin Resistance (EDIR)	0.9 ± 0.4	1.0 ± 0.5	0.9 ± 0.3	0.044
Empirical Lifestyle Index for Hyperinsulinemia (ELIH)	0.16 (-0.49—0.58)	0.32 (-0.06—0.73)	0.07 (-0.69—0.46)	< 0.001
Empirical Lifestyle Index for Insulin Resistance (ELIR)	4.4 ± 2.6	5.4 ± 3.1	3.9 ± 2.1	< 0.001

^a^All values are reported as mean ± standard deviation (SD) for quantitative variables and as number (percentage) or percentage for qualitative variables

^b^Differences in demographic characteristics and nutritional intakes between the two groups of people with non-alcoholic fatty liver disease (NAFLD) and non-NAFLD individuals were assessed using an independent sample t-test for quantitative variables and a Chi-square test for qualitative variables

Participants' characteristics are also expressed across the tertiles of EDIH and ELIH in Tables 2 and 3, respectively. According to the results reported in Table 2, participants in the highest tertile of the EDIH are significantly more male and have a lower mean of physical activity than those in the lowest tertile of EDIH (*P* < 0.05). However, the mean of age, BMI, percentage of smoking, percentage of academic education, and SES were not different across tertiles of EDIH (*P* > 0.05). The intakes of energy, protein, fats, red meat, processed meats, margarine, French fries, chicken, butter, fish, high-energy beverages, eggs, and tomatoes significantly increased across tertiles of EDIH (*P* < 0.05), whereas dietary intakes of carbohydrates and whole fruit were significantly decreased across tertiles of EDIH (*P* < 0.05). The intakes of high-fat dairy, low-fat dairy, coffee, and green leafy vegetables were not different across tertiles of EDIH (*P* > 0.05).

**Table 2:** Baseline characteristics and dietary intakes of the participants in the tertiles empirical dietary index for hyperinsulinemia (EDIH)

Variables	Tertiles of the score			*P*-value
	T1 (*n* = 136)	T2 (*n* = 105)	T3 (*n* = 136)	
Age (year)	42.7 ± 8.2	41.4 ± 7.2	41.4 ± 7.2	0.244
Sex (male, %)	47.9	46.7	62.5	0.017
BMI (Kg/m^2^)	27.5 ± 2.2	27.1 ± 2.3	27.8 ± 2.2	0.251
Physical activity (MET.min/Wk.)	1492 ± 858	1672 ± 1071	1327 ± 689	0.049
Smoking (yes, %)	2.5	8.6	5.9	0.140
Household dimension (≤ 4 people), n (%)	75 (63.0)	69 (65.7)	98 (72.1)	0.286
Education level (bachelor and higher), n (%)	43 (36.1)	53 (50.5)	64 (47.1)	0.072
Housing status (landlord), n (%)	77 (64.7)	66 (62.9)	98 (72.1)	0.236
Socio-economic status (SES), n (%)				
Poor	49 (41.2)	41 (39.0)	48 (35.3)	0.189-
Medium	46 (38.7)	37 (35.2)	51 (37.5)	-
High	24 (20.2)	27 (25.7)	37 (27.2)	-
Energy intake (kcal/day)	2077 ± 604	2217 ± 643	2445 ± 649	< 0.001
Carbohydrates (% of energy)	57.3 ± 6.7	57.0 ± 5.8	54.5 ± 7.1	< 0.001
Protein (% of energy)	13.0 ± 2.0	13.4 ± 2.1	13.8 ± 2.7	0.015
Fat (% of energy)	29.7 ± 6.7	29.6 ± 5.7	31.7 ± 7.1	0.009
Empirical dietary index for hyperinsulinemia (EDIH) components				
Red meat (portion per day)^a^	0.09 (0.05—0.14)	0.12 (0.06—0.17)	0.16 (0.08—0.25)	< 0.001
Processed meat (portion per day)^a^	0.010 (0.002—0.030)	0.014 (0.002—0.033)	0.020 (0.006—0.055)	0.002
Margarine (portion per day)	0 (0—0)	0 (0—0)	0 (0—0.1667)	< 0.001
Chicken (portion per day)^a^	0.09 (0.08—0.17)	0.11 (0.09—0.21)	0.18 (0.09—0.34)	< 0.001
Butter (portion per day)	0.05 (0—0.17)	0.33 (0—0.71)	1.43 (0.33—2.86)	< 0.001
French fries (portion per day)^b^	0.01 (0—0.03)	0.02 (0.01—0.04)	0.04 (0.08—0.06)	< 0.001
Fish (portion per day)^b^	0.05 (0.03—0.10)	0.05 (0.03—0.12)	0.07 (0.04—0.13)	0.033
High-energy beverages (daily portion)	0.04 (0.00—0.05)	0.04 (0.00—0.08)	0.04 (0.00—0.16)	0.001
Tomatoes (portion per day)	0.62 ± 0.42	0.88 ± 0.54	1.14 ± 0.82	< 0.001
Low fat dairy (portion per day)	0.92 ± 0.73	1.01 ± 0.85	1.00 ± 0.77	0.477
Eggs (portion per day)	0.13 (0.06—0.27)	0.27 (0.13—0.4015)	0.27 (0.13—0.47)	< 0.001
Coffee (portion per day)	0.68 (0—8.33)	0.69 (0—8.33)	0.70 (0—8.33)	0.363
Whole fruit (portion per day)	3.7 ± 1.5	3.4 ± 1.2	3.2 ± 1.1	0.008
High-fat dairy (portion per day)	1.2 ± 1.1	0.9 ± 0.5	1.00 ± 0.6	0.056
Green leafy vegetables (portion per day)	0.27 (0.16—0.52)	0.25 (0.14—0.53)	0.26 (0.15—0.49)	0.580

All values are reported as mean ± standard deviation or median (IQR) for quantitative variables and as number (percentage) or percentage for qualitative variables

^a^Each portion = 5 oz (142 g)

^b^Each portion = 4 oz (113 g)

**Table 3:** Baseline characteristics and dietary intakes of the participants in the tertiles empirical lifestyle index for hyperinsulinemia (ELIH)

Variables	Tertiles of the score			P-trend
	T1 (*n* = 103)	T2 (*n* = 118)	T3 (*n* = 139)	
Age (year)	40.7 ± 7.1	42.5 ± 7.6	42.0 ± 7.7	0.146
Sex (male, %)	51.5	54.2	53.2	0.806
BMI (Kg/m^2^)	26.6 ± 2.0	27.0 ± 1.9	28.4 ± 2.3	< 0.001
Physical activity (MET.min/Wk.)	2595 ± 796	1317 ± 221	798 ± 285	< 0.001
Smoking (yes, %)	6.8	4.2	5.8	0.778
Education level (bachelor and higher), n (%)	54 (52.4)	48 (40.7)	58 (41.7)	0.154
Socio-economic status (SES), n (%)				
Poor	43 (41.7)	42 (35.6)	53 (38.1)	0.362
Medium	37 (35.9)	50 (42.4)	47 (33.8)	
High	23 (22.3)	26 (22)	39 (28.1)	
Energy intake (kcal/day)	2209 ± 629	2205 ± 572	2336 ± 721	0.162
Carbohydrates (% of energy)	58.4 ± 7.0	57.3 ± 7.4	57.3 ± 6.8	0.182
Protein (% of energy)	13.7 ± 2.0	13.7 ± 2.4	13.8 ± 2.6	0.819
Fat (% of energy)	30.4 ± 6.7	31.7 ± 7.1	31.4 ± 6.5	0.227
Empirical lifestyle index for hyperinsulinemia (ELIH) components				
Margarine (portion per day)	0.0 (0.0—0.012)	0.0 (0.0 – 0.0)	0.0 (0.0 – 0.0)	0.145
Butter (portion per day)	0.17 (0—0.71)	0.25 (0.01—1.15)	0.71 (0.07—1.79)	< 0.001
Red meat (portion per day) ^a^	0.12 (0.06—0.18)	0.10 (0.06—0.18)	0.12 (0.07—0.22)	0.728
Fruit juices (portion per day)	0.04 (0.02—0.10)	0.03 (0.01—0.10)	0.03 (0.01—0.07)	0.241
Coffee (portion per day)	0.00 (0—0.04)	0.00 (0—0.04)	0.00 (0—0.04)	0.357
Whole fruit (portion per day)	3.59 ± 1.33	3.40 ± 1.29	3.31 ± 1.25	0.090
High-fat dairy (portion per day)	1.0 ± 0.58	1.07 ± 1.06	1.00 ± 0.62	0.820
Snacks (portion per day)	0.13 (0.02—0.20)	0.08 (0.01—0.15)	0.09 (0.01—0.16)	0.238
Salad dressing (portion per day)	0.09 (0.03—0.25)	0.08 (0.03—0.20)	0.11 (0.03—0.22)	0.441

All values are reported as mean ± standard deviation or median (IQR) for quantitative variables and as number (percentage) or percentage for qualitative variables

^a^Each portion = 5 oz (142 g)

According to Table 3, participants in the highest tertile of the ELIH score significantly had lower physical activity and higher BMI than those in the lowest tertile of the ELIH score (*P* < 0.05). However, there were no significant differences in mean age, gender distribution, percentage of smoking, percentage academic education, and SES across tertiles of ELIH (*P* > 0.05). The dietary intakes of energy, fats, protein, and carbohydrates were not different across tertiles of ELIH (*P* > 0.05). Regarding the components of the ELIH score, our findings indicate that the intake of butter significantly increased across tertiles of ELIH (*P* < 0.05), however, the intakes of margarine, red meat, fruit juices, whole fruit, coffee, high-fat dairy, snacks, and salad dressing were not different across tertiles of ELIH (*P* > 0.05).

The odds of NAFLD across the tertiles of EDIH and ELIH are shown in Table 4. In the age and sex-adjusted model, no significant association was found between the higher score of EDIH and odds of NAFLD (OR:1.23; 95% CI: 0.72–2.10), (P for trend = 0.235). Also, based on the multivariable-adjusted model, after controlling for age, sex, energy intake, smoking, physical activity, and SES score, there was no significant association between EDIH and odds of NAFLD (OR: 0.91; 95% CI: 0.51–1.62), P for trend = 0.895). In the age and sex-adjusted model, the odds of NAFLD were increased across tertiles of ELIH score (OR = 2.74; 95%CI: 1.51 – 4.96, P for trend = 0.001). Also, based on the multivariable-adjusted model, after adjusting age, gender, SES score, energy intake, and smoking, the odds of NAFLD was increased across tertiles of ELIH (OR = 2.70; 95%CI: 1.46–5.01, P for trend = 0.002).

**Table 4:** Odds ratio (95% confidence interval) of non-alcoholic fatty liver disease among the study participants in the tertiles of the empirical dietary index for hyperinsulinemia (EDIH) and empirical lifestyle index for hyperinsulinemia (ELIH) scores

	Tertiles of the score			P-trend
	T1 (Ref.)	T2	T3	
**EDIH**				
** Median score**	0.04	0.14	0.33	
** Case/control**	39/80	25/80	56/80	
** Crude model**	1	0.64 (0.36—1.16)	1.44 (0.86—2.40)	0.064
** Model 1 **^**a**^	1	0.63 (0.34—1.15)	1.23 (0.72—2.10)	0.235
** Model 2 **^**b**^	1	0.52 (0.26—0.99)	0.91 (0.51—1.62)	0.895
**ELIH**				
**Median score**	-1.05	0.09	0.69	
**Case/control**	23/80	38/80	59/80	
**Crude model**	1	1.65 (0.90—3.02)	2.58 (1.45—4.55)	0.001
**Model 1 **^**a**^	1	1.69 (0.91—3.16)	2.74 (1.51—4.96)	0.001
**Model 2 **^**c**^	1	1.72 (0.90—3.28)	2.70 (1.46—5.01)	0.002

^a^Adjusted for age and sex

^b^Adjusted for the model 1 and energy, socio-economic status, smoking, and physical activity

^c^Adjusted for model 1 and energy, socio-economic status, smoking

## Discussion

In the present study, our findings indicated that a higher score of ELIH can be related to an increased odds of NAFLD independent of the effects of various potential confounding factors. However, there was no significant association between the higher score of EDIH and the odds of NAFLD.

Studies on the association of EDIH with the developing NAFLD worldwide are limited to two studies conducted among Asian peoples [12, 23]. Sohouli et al. reported that a diet with higher insulinemic potential may be associated with an increased odds of NAFLD [23]. Also, a population-based cohort study conducted on the USA population revealed that individuals with a higher score of EDIH are more prone to the prevalence of both steatosis and fibrosis [12]. However, contrary to the results mentioned in the previous study, our research findings on the relationship between EDIH and odds of NAFLD were not statistically significant. Various reasons can explain this difference in results. The non-significant results of the current study can be due to the low intake of EDIH components in our study population and, consequently, its low score estimation. Also, our participants' EDIH score range was narrow, so the difference in EDIH scores of individuals in the third tertile of EDIH was not very remarkable compared to those in the first tertile of EDIH. The different people studied can be one of the causes of this inconsistency in findings. Also, EDIH is a dietary insulinemic index determined based on intakes of a wide range of dietary determinants; the interaction of anti/pro-insulinemic foods may attenuate EDIH's ability to predict hyperinsulinemia and NAFLD odds. Furthermore, the small sample size and the low power of the study can be an important influencing factor in the non-significance of the results on the relationship between EDIH and odds of NAFLD.

To the best of our knowledge, up to now, there is no study on the association between ELIH and the odds of NAFLD. However, our findings on the association of ELIH with the odds of NAFLD are comparable with the results of some previous studies that have shown a significant association between the insulinemic potential of lifestyle and increased risk for some other chronic diseases such as type 2 diabetes [8], insulin resistance [7], and cancers [13, 14]. Farhadnejad et al. reported that greater adherence to a lifestyle with a higher score of ELIH score was associated with an increased risk of type 2 diabetes [8]. Also, Mokhtari et al. revealed that a higher score of ELIH is related to an increased risk of insulin resistance, insulin insensitivity, and hyperinsulinemia [7]. Also, the study by Tabung et al. [13] and Wang et al. [14] have suggested that the higher insulinemic potential of diet or lifestyle can be linked to a higher risk of cancers in both men and women. Therefore, the results of our study, as well as previous evidence from studies on other populations, support that a lifestyle with a higher score of EDIH, indicating the insulinemic potential of the combined three important lifestyle factors including BMI, physical activity, and dietary pattern, has a high power in predicting the risk of chronic diseases such as NAFLD.

In the current study, the ELIH score had greater power in the prediction of the development of NAFLD than the EDIH score; these results were to be expected because the determinants of the ELIH score (BMI, physical activity, and diet) individually are the main predictors of the NAFLD odds. Indeed, the cooperative contributions of major lifestyle-related factors, including obesity, physical inactivity, and insulinemic dietary pattern to insulin secretion and its metabolism, showed a remarkable relationship with NAFLD compared to EDIH as an alone dietary insulinemic score. Therefore, the insulinemic potential created by ELIH components in individuals with a greater score of ELIH leads these subjects much more prone to increased NAFLD odds.

Our results on the association of ELIH with the odds of NAFLD can be explained by the evidence of previous research indicating the possible link between modifiable factors including adiposity, diet, and physical as components of the ELIH with hyperinsulinemia and odds of NAFLD. It was previously reported that obesity is related to hypo-adiponectinemia, insulin resistance, and hyperinsulinemia [24] and may increase NAFLD odds [25]. Also, low physical activity levels and poor dietary patterns are potentially higher linked to BMI and fat mass and a higher risk of insulin-related disorders and consequently can increased odds of NAFLD [26–28]. According to previous reports, unhealthy dietary patterns such as the western diet, which can have high insulin secretion ability, and an unhealthy lifestyle, may play an important role in developing chronic diseases such as NAFLD [29]. However, a low insulinemic diet rich in whole fruits, vegetables, and leafy green vegetables (with high content of fiber, calcium, magnesium, potassium) and poor in red meat, processed meats, a simple sugar, refined cereals is related to lower risk of hyperinsulinemia [6, 15].

This study has some important strengths. To the best of our knowledge, this is the first study to assess lifestyle insulinemic potential concerning the NAFLD odds. Also, we used valid and reliable questionnaires for the assessment of nutritional data and physical activity levels in participants. The current study had some limitations. First, because of the study’s case–control design of this study, we could not discover the causal relationships. Second, although a validated FFQ was used to estimate nutritional intakes, the possible measurement error is unavoidable. Third, we used ultrasonography for the diagnosis of NAFLD, however, liver biopsy is the gold standard for detection of NAFLD, and also magnetic resonance imaging (MRI) technique is more accurate; it should be noted, this defect can be neglected because today, due to the limitations and complications of biopsy and high cost and low availability of MRI techniques, using noninvasive methods ultrasonography is more reliable and applicable in clinical practice [30]. Considering that our study was a case–control study, recall bias and selection bias were two inherent and unavoidable limitations of our study. Furthermore, despite the controlling of various confounders, we cannot eliminate all potential confounders, and the effects of some residual confounders may have occurred.

## Conclusions

In conclusion, our results suggested that the higher insulinemic potential of lifestyle, determined by a poor diet and inappropriate levels of physical activity and BMI, maybe associated with an increased odds of NAFLD in Iranian adults. However, no significant association was observed between a higher score of EDIH and odds of NAFLD. Further observational studies are recommended to investigate the possible role of EDIH and ELIH in the development of NAFLD among other populations.

## Data Availability

The datasets analyzed in the current study are available from the corresponding author on reasonable request.

## Abbreviations

BMI: Body mass index
CI: Confidence interval
CVD: Cardiovascular disease
EDIH: Empirical dietary index for hyperinsulinemia
ELIH: Empirical lifestyle index for hyperinsulinemia
FFQ: Food frequency questionnaire
FCT: Food Composition Table
IPAQ: International Physical Activity Questionnaire
IR: Insulin resistance
METs: Metabolic Equivalents
MRI: Magnetic resonance imaging
NAFLD: Non-alcoholic fatty liver disease
OR: Odds ratio
SES: Socioeconomic status
SPSS: Statistical Package Software for Social Science
USDA: United States Department of Agriculture
USG: Ultrasonography
